# Physical activity, CVD, and older adults

**DOI:** 10.18632/aging.101942

**Published:** 2019-05-01

**Authors:** Richard A. Ferraro, Vincent A. Pallazola, Erin D. Michos

**Affiliations:** 1 Johns Hopkins Ciccarone Center for the Prevention of Cardiovascular Disease, Baltimore, MD 21287, USA

**Keywords:** physical activity, cardiovascular disease prevention, elderly

Physical activity (PA) has long been an underutilized point of intervention in cardiovascular disease (CVD) prevention, with an estimated 10% of premature mortality and $117 billion healthcare dollars annually linked to inadequate PA [1]. Women, minorities, and older adults are more likely to not meet recommended PA levels compared to their respective counterparts [2]. The recently updated 2018 PA Guidelines for Americans put forth by the Department of Health and Human Services noted specific benefits of PA in the aging population, such as a reduction in the risk of falls, injuries, and dementia, including Alzheimer Disease [1]. Given that CVD is the number one cause of mortality among older adults and a significant source of healthcare spending, lifestyle interventions like PA in the prevention of CVD have become paramount.

A leading feature of the 2018 PA guidelines is the recommendation that adults, including older adults, spend at least 150-300 minutes per week on moderate-intensity aerobic exercise, or 75-150 minutes of vigorous exercise, as defined as 3-5.9 metabolic equivalents (METs) or ≥6 METs, respectively [1]. Importantly, the guidelines highlight that while 150-300 minutes of moderate-intensity may be the goal, benefits are also seen at even lower degrees of PA [1]. This is supported by recent work in 63,591 adults highlighting that even 1-2 sessions per week of PA at <150 minutes of moderate-intensity led to reductions in all-cause mortality (Hazard Ratio (HR) 0.66, Confidence Interval (CI): 0.62-0.72) and CVD mortality (HR 0.60, 95% CI: 0.52-0.69) as compared to inactive participants [3]. Similar endorsements for at least 150 minutes/week of moderate-to-vigorous PA were also given a strong recommendation in the 2019 Primary Prevention Guidelines from the American College of Cardiology (ACC)/American Heart Association (AHA) [4]. The ACC/AHA guidelines also state that for adults who are unable to meet recommended PA levels, engaging in any amount of moderate-to-vigorous intensity PA, even if less than recommended, could reduce their CVD risk [4]. Additionally, reductions in sedentary behavior are also reasonable for CVD risk reduction [4].

Understanding that even limited amounts of PA can still be beneficial is of particular importance in the aging population, where comorbidities may make 150 minutes of moderate-intensity or 75 minutes of vigorous-intensity PA more difficult, or even unsafe, to achieve. The guidelines highlight this point specifically, suggesting that those unable to perform 150 minutes of moderate-intensity activity should be as physically active as any limiting medical conditions allow [1]. Even walking/biking within the confines of a commute exhibited CVD benefit in a meta-analysis, with an 11% reduction in CVD risk (Risk ratio (RR) 0.89, CI: 0.81-0.98); replacing sitting with light activity is additionally known to reduce mortality [5,6]. A further benefit in the updated guidelines is the removal of a minimum amount of PA in a single setting (known as “bouts”) required to contribute toward daily total exercise goals (previous guidelines stated 10 minutes as a minimum increment), such that now all PA throughout the day counts toward the 150-minute weekly total [1,4].

Furthermore, there does not appear to be an upper bound on the CVD benefits derived from PA in those able to exceed PA recommendations. As cited in the guidelines, there is strong evidence of an inverse dose-response relationship between moderate and/or vigorous PA and CVD mortality [1,4]. Therefore, while reaching the recommended 150 minutes of moderate-intensity PA associated with a 40% reduction in CVD mortality, those going beyond this level to at least 3-5 times the current recommendations continued to exhibit a CVD mortality benefit [1].

An area specifically highlighted by the guidelines with respect to older patients is the importance of multi-component activity that includes aerobic in addition to balance and weight-training exercise [1]. This has displayed relevance not only in primary prevention, but also in older patients with known CVD, with studies in resistance training and complementary exercises in older adults exhibiting improved mobility as compared to controls. It is important to note that while multi-component exercise is broadly endorsed by the guidelines in older patients, due to a small number of studies on CVD-specific effects, the 2018 PA guideline committee assigned only a limited evidence recommendation for the addition of multi-component exercise to improvements in CVD risk [1]. The utility of categorizing these activities as either low, moderate, or high-intensity activities in older populations, where relative intensity may serve as a better marker than the absolute intensity of the activity, has yet to be delineated.

The benefits of PA on CVD risk and mortality are clear and growing. In older adults aged ≥70, higher fitness was associated with improved survival; whereas there was no association of traditional risk factors and survival in this older cohort [7]. As the updated 2018 PA guidelines highlight, older patients are one of the populations to receive the greatest benefit from PA interventions [1]. While meeting or exceeding guideline recommendations of 150 minutes of moderate-intensity or 75 minutes vigorous-intensity activity is the goal, even modest increases in activity can lead to marked reduction in CVD morbidity, mortality, and healthcare spending. Further research in needed on the contribution of light activity, the effect of multi-component exercise on CVD risk and mortality, and greater precision on the risk/benefit of those with chronic conditions exerting themselves. Regardless, the data to date are robust on the cardiovascular benefits of PA derived by aging patients, and the updated PA guidelines act as an important step forward in promoting better outcomes for this patient population.
